# Lipoprotein(a) and plaque progression: insights from serial coronary computed tomography angiography and quantitative plaque assessment

**DOI:** 10.3389/fcvm.2026.1699503

**Published:** 2026-02-05

**Authors:** Xiaofang Chen, Yonghong Zheng, Shaowei Lin, Xiaomin Dai, Yang Chen, Shun Yu

**Affiliations:** 1 Department of Radiology, Shengli Clinical Medical College of Fujian Medical University, Fujian Provincial Hospital, Fuzhou University Affiliated Provincial Hospital, Fuzhou, Fujian, China; 2 Department of Epidemiology and Health Statistics, School of Public Health, Fujian Medical University, Fuzhou, Fujian, China; 3 School of Medical Imaging, Fujian Medical University, Fuzhou, Fujian, China; 4 Fujian Provincial Key Laboratory of Medical Big Data Engineering, Fuzhou, Fujian, China

**Keywords:** atherosclerosis, coronary computed tomography angiography (CCTA), lipoprotein(a), plaque progression, plaque quantitative analyses

## Abstract

**Background:**

Lipoprotein(a) [Lp(a)] is a well-established independent risk factor for cardiovascular disease. However, the long-term effects of Lp(a) on coronary plaque phenotype remain unclear.

**Objective:**

To explore the potential association between Lp(a) levels and coronary plaque volume, composition, and progression using coronary computed tomography angiography (CCTA).

**Methods:**

Patients with available data for Lp(a) and underwent baseline CCTA examinations between January 2009 to December 2015 and subsequently underwent a follow-up coronary CTA were retrospectively enrolled. Quantitative CCTA analyses measured plaque length, total plaque volume and composition volume. Patients were categorized into an elevated Lp(a) group (≥30 mg/dL) and a normal Lp(a) group (<30 mg/dL). The association between Lp(a) and baseline plaque characteristic and progression were investigated in linear mixed-effects models adjusted for clinical factors. Subgroup analyses were also conducted.

**Results:**

Among 453 patients (mean age 64.7 years, 77.7% male) with a median follow-up of 6.15 years. elevated Lp(a) was linked to higher baseline plaque burden (all *p* < 0.001) and accelerated LAP volume progression (*β* = 0.55 mm^3^/year, 95% CI: 0.04–1.06; *p* = 0.036) after adjusting for confounders. In addition, patients with diabetes, female gender, family history of CAD, or aged <60 years and with normal lipid profiles showed higher progression in total plaque volume and LAP, fibro-fatty, and fibrous components. Increased calcification volume progression was also seen in those with diabetes, female gender, smoking, drinking, or normal LDL-C levels. The association between Lp(a) and calcification progression was more pronounced in statin users.

**Conclusions:**

Elevated Lp (a) level was associated with high coronary artery plaque burden at baseline and rapid progression of LAP at follow-up. Lp(a) may serve as a significant residual risk factor in seemingly “low-risk” populations.

## 1 Introduction

Coronary artery disease (CAD) remains the leading cause of global mortality, accounting for 16% of all deaths worldwide in 2019 according to the Global Burden of Disease Study (1). While statins are central to reducing low-density lipoprotein cholesterol (LDL-C) and preventing atherosclerotic cardiovascular disease (ASCVD) (2), many high-risk individuals continue to experience plaque progression and adverse events, indicating a residual cardiovascular risk.

Lipoprotein(a) [Lp(a)] has reemerged as a key factor in CAD pathogenesis, distinguished from LDL by its apolipoprotein(a) component rich in oxidized phospholipids (OxPL) that promote atherogenesis, thrombosis, and inflammation (3). Numerous large-scale prospective studies (4, 5) have confirmed Lp(a) as a causal risk factor for cardiovascular disease, independent of LDL-C and other traditional risk factors. Elevated Lp(a) levels are associated with increased atherosclerotic plaque burden and adverse plaque features, as demonstrated by intravascular ultrasound (IVUS) and optical coherence tomography (OCT) studies (6, 7). However, these invasive imaging modalities limit broad clinical application.

Coronary computed tomography angiography (CCTA) is a valuable non-invasive tool for diagnosing and evaluating CAD, offering detailed visualization of coronary arteries and plaque characteristics (8). It enables assessment of plaque composition—including calcified, non-calcified, and low-density necrotic core components—with high reproducibility, facilitating effective disease monitoring (9). Quantitative CCTA (QCT) analysis further allows identification of low-attenuation plaques (LAP, ≤30 Hounsfield Unit, HU), which are strongly associated with acute coronary events (10, 11).

In summary, the long-term relationship between Lp(a) and coronary artery plaque progression remains incompletely understood. CCTA offers distinct advantages in the non-invasive assessment and longitudinal monitoring of coronary plaque changes. This study aims to investigate the association between Lp(a) levels and dynamic coronary plaque changes using serial quantitative CCTA-based plaque quantification.

## 2 Materials and methods

### 2.1 Study population

Patients who underwent baseline CCTA at Fuzhou University Affiliated Provincial Hospital or its South Branch between January 2009 and December 2015, and subsequently had follow-up CCTA, were retrospectively enrolled in this study. Exclusion criteria were as follows:(1) all lesion with <25% diameter stenosis in major epicardial arteries (luminal diameter ≥2 mm) on both baseline and follow-up CCTA; (2) absence of serum Lp(a) measurement within one month before or after baseline CCTA; (3) history of acute coronary syndrome (ACS); (4) history of coronary revascularization at baseline or having undergone revascularization between serial CCTA examination; (5) poor image quality of CCTA precluding accurate analysis; (6) presence of other comorbidities, such as aortic dissection, valvular heart disease, congenital heart disease, cardiomyopathy, or Takayasu arteritis.

### 2.2 Lp(a) measurement and study population stratification

Plasma Lp(a) concentrations were measured using a particle-enhanced immunoturbidimetric assay (Roche Cobas 8000) and reported in mg/dL. As multiple studies have shown that Lp(a) levels remain stable over a lifetime in more than 90% of adults (12), they were considered constant throughout the study period. The baseline Lp(a) level was used in this study.

According to Guidelines for the Prevention and Treatment of Dyslipidemia in Chinese Adults (13), 30 mg/dL was selected as the cutoff value. Based on serum Lp (a) levels, the study population was stratified into two groups: an elevated Lp(a) group (≥30 mg/dL) and a normal Lp(a) group (<30 mg/dL).

To assess the robustness of our findings across different Lp(a) thresholds, a sensitivity analysis was also conducted using a higher cutoff of 50 mg/dL, which has been used in prior epidemiological and clinical studies.

### 2.3 CCTA imaging acquisition

At baseline, all patients underwent combined coronary artery calcium scoring (CACS) and CCTA using a first-generation dual-source CT scanner (SOMATOM Definition, Siemens Healthineers, Germany). At follow-up, some patients underwent combined CACS and CCTA using a third-generation dual-source CT (SOMATOM Force, Siemens Healthineers, Germany). Before scanning, patients were instructed on breath-holding techniques to minimize respiratory motion artifacts. The scan range extended from the carina of the trachea to 1 cm below the diaphragmatic surface of the heart, including the entire heart from the left apex to the right border. Baseline CCTA was performed using a retrospective electrocardiogram (ECG)-gated CCTA protocol triggered between 30% to 80% of the R-R interval. Follow-up CCTA in some patients used a prospective ECG-gated CCTA protocol triggered at 70% of the R-R interval. Contrast enhancement was achieved by injecting 50–60 mL of iopromide (350 mg I/mL) through the right antecubital vein at 5 mL/s, followed by a 50 mL saline bolus chaser at the same flow rate. The parameters and scanning modes are detailed in the Online Supplementary Materials.

### 2.4 AI-QCT analysis

The raw CCTA data (baseline and follow-up) were electronically transferred to an artificial intelligence (AI)-based coronary-specific analysis software (CoronaryDoc, ShuKun Technology, Beijing, China) (14, 15) for image analysis. This software, approved by the National Medical Products Administration (NMPA), uses deep learning algorithms to automatically identify and analyze plaque features in coronary vessels and has been trained on large multicenter CCTA datasets. Previous studies have shown that CoronaryDoc can reliably detect and classify coronary atherosclerotic plaques and provides quantitative plaque measurements (16).

In our workflow, the software automatically performs coronary tree extraction, lumen and vessel wall segmentation, and plaque quantification based on predefined HU thresholds, using a fully automated and deterministic pipeline without manual editing of contours. For a given CCTA dataset, repeated analyses by different observers or at different time points therefore yield identical quantitative outputs. A trained observer, blinded to patients' Lp(a) levels and other clinical data, independently reviewed all AI-generated results to ensure anatomical plausibility and corrected only obvious segmentation failures, when necessary, while the volumetric plaque parameters were obtained directly from the software.

All coronary artery segments with a diameter ≥2 mm were analyzed using a modified 17-segment American Heart Association model (17). Each segment was evaluated for the presence or absence of coronary atherosclerosis. Coronary plaque was defined as any tissue ≥1 mm^3^ within or adjacent to the lumen that could be discriminated from the surrounding structures and identified in at least two imaging planes (10). All lesions with ≥25% diameter stenosis at baseline or follow-up CCTA imaging were included in the analysis.

For each coronary lesion, plaque volume (PV) was calculated, and total plaque volume (TPV) was obtained by summing PVs across all lesions. Plaque composition volumes were determined using predefined HU cutoff values of CCTA (18): (1) dense calcium is assigned to HU densities greater than 350 HU, and −30–350 HU for non-calcified plaque; (2) non-calcified plaque was further classified in fibrous component (131–350 HU), fibro-fatty component (31–130 HU) and LAP (−30–30 HU). Plaque volumes for each category were assessed across all coronary segments and summed to obtain patient-level TPVs. The percentage of each plaque component was calculated by its volume divided by the TPV.

The software automatically generated the following quantitative parameters: percentage diameter stenosis (%DS), plaque length (PL), minimal lumen area (MLA), calcified volume (CV), fibrotic volume (FV), fibro-fatty volume (FFV), LAP volume (LAPV). The non-calcified volume (NCV, equivalent to LAPV + FFV + FV), along with corresponding volume ratios (CVR, FVR, FFVR, LAPVR). Plaque diffuseness was defined as the number of lesions with ≥25% diameter stenosis. For longitudinal comparison, baseline and follow-up CCTAs were analyzed side-by-side. Coronary lesions were co-registered using fiduciary landmarks, including distance from the ostium and branch vessels. A trained observer, blinded to patients' Lp(a) levels and other clinical data, independently analyzed all CCTA results.

### 2.5 Clinical information recorded

Patient characteristics, including serological examinations, medications and clinical risk factors, were collected by reviewing electronic medical records and conducting telephone interviews at each CCTA examination.

### 2.6 Statistical analysis

Quantitative variables were tested for normality using the Shapiro–Wilk test. Data are presented as mean (SD) for normally distributed variables or median with interquartile range (IQR) for non-normally distributed data. Categorical variables are expressed as absolute numbers and percentages. To compare clinical features of two groups, The Student t test, Mann–Whitney *U* test, or *χ*^2^ test was used, as appropriate.

The association between Lp(a) and plaque volumes over time was assessed using linear mixed-effects regression models with a random intercept to account for within-patient clustering. In these models, an interaction term between time and Lp(a) group was included to assess the effect of Lp(a) on plaque progression. The multivariable linear mixed-effects models were adjusted for age, sex, clinical risk factors (hypertension, hypercholesterolemia, diabetes, smoking status, family history of CAD), LDL-C, high-density lipoprotein cholesterol (HDL-C), total cholesterol level (TC), CACS, and statin used. Subgroup analyses were conducted after categorizing the patients according to sex, age, hypertension, diabetes mellitus, smoking status, family history of CAD, statin used, TC, LDL-C level, and CACS.

## 3 Results

### 3.1 Baseline clinical and imaging characteristics

**Figure 1:**
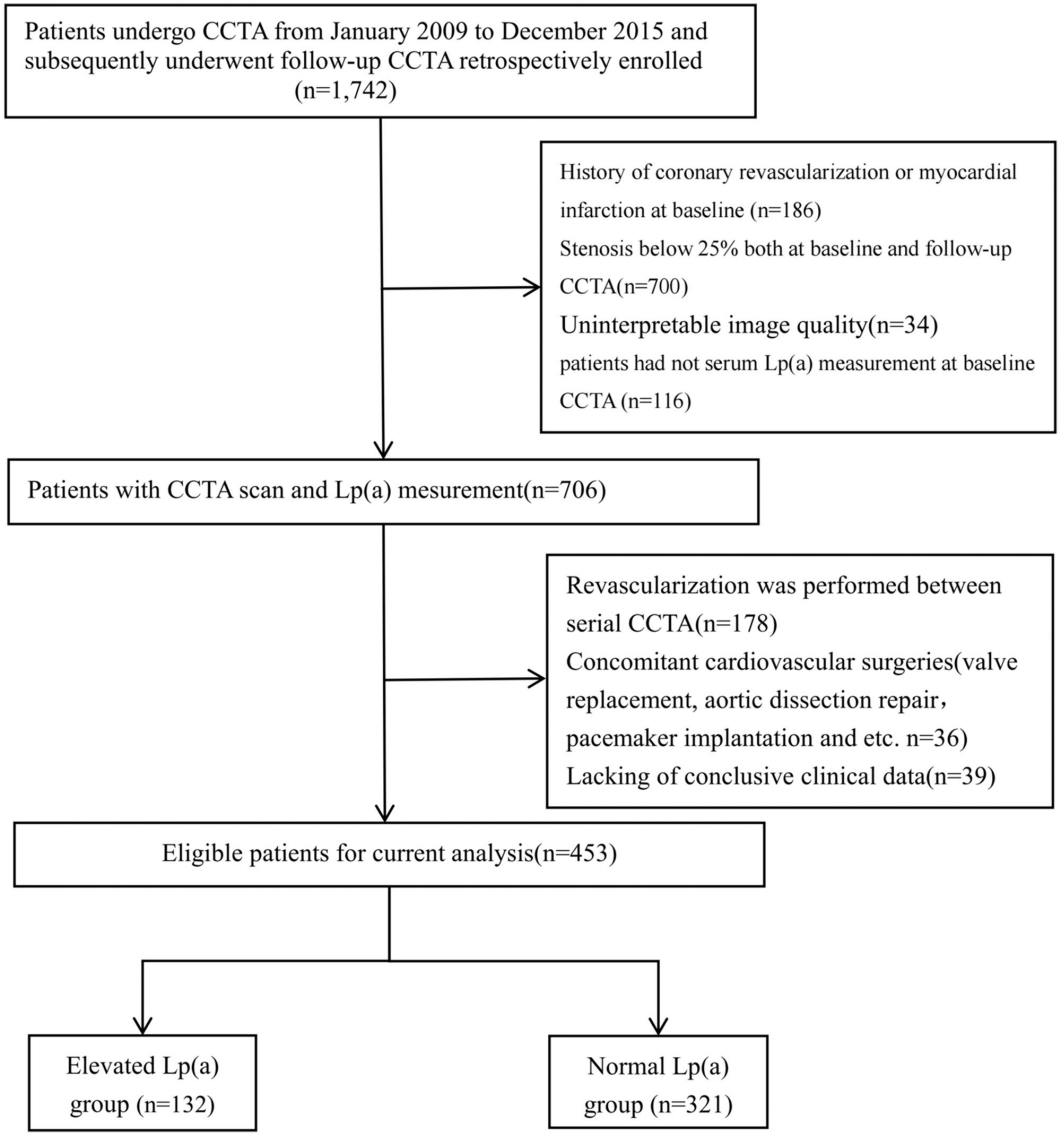
Study flow. Patients undergone CCTA From January 2009 to December 2015 and subsequently underwent follow-up CCTA were retrospectively enrolled. After screening of enrolment criteria and image quality, 453 patients were finally analyzed.

The study flow chart is shown in Figure 1. According to the inclusion and exclusion criteria, 453 patients (mean age 64.7 ± 8.8 years; 77.7% male) were enrolled. All patients underwent at least two CCTA examinations over a median interval of 6.15 years (Q1–Q3: 4.10–8.25). The median TPV for the entire coronary tree was 84.47 mm^3^ (Q1–Q3: 42.37–153.94). Lp(a) level showed a right-skewed distribution, with a median of 14.17 mg/dL (Q1–Q3: 6.72–32.49) (Supplementary Figure S1).

Baseline clinical and imaging characteristics stratified by Lp(a) group are summarized in Table 1. Compared with the normal Lp(a) group, the elevated Lp(a) group exhibited a higher prevalence of atherosclerotic plaques (2.51 ± 1.52 vs. 1.92 ± 1.18; *p* < 0.001), a greater proportion of current smokers and individuals with a family history of CAD, and a shorter CCTA follow-up interval [5.18 (3.33–7.27) vs. 6.52 (4.67–8.59) years; *p* < 0.001]. Although the prevalence of diabetes mellitus was similar between groups, patients with elevated Lp(a) had significantly higher HbA1c levels (6.96 ± 1.62 vs. 6.53 ± 1.19; *p* = 0.007). They also had higher LDL-C (3.08 ± 0.91 vs. 2.82 ± 1.05 mmol/L; *p* = 0.011) and total cholesterol (4.74 ± 1.06 vs. 4.50 ± 1.00 mmol/L; *p* = 0.027) levels. No significant differences were observed in age, sex, hypertension, dyslipidemia, Drink, HDL-C, triglycerides, hs-CRP, or medication use at baseline (all *p* > 0.05).

**Table 1:** Baseline characteristics comparison according to Lp(a) levels at baseline CCTA.

Variables	Over all (*n* = 453)	Elevated Lp(a) group (*n* = 132)	Normal Lp(a) group (*n* = 321)	*P* value
Age, years	64.68 ± 8.80	63.40 ± 9.28	65.21 ± 8.56	0.055
Male, *n* (%)	352 (77.70%)	242 (75.39%)	110 (83.33%)	0.085
Heart Rate, BPM	75.25 ± 10.95	74.97 ± 12.14	75.36 ± 10.44	0.744
Respiratory Rate, BPM	19.93 ± 6.59	19.86 ± 5.20	19.96 ± 7.09	0.877
SBP, mmHg	136.04 ± 17.51	135.16 ± 20.55	136.40 ± 16.12	0.537
DBP, mmHg	75.92 ± 10.56	76.18 ± 11.70	75.81 ± 10.07	0.752
Plaque diffuseness, *n*	2.09 ± 1.32	2.51 ± 1.52	1.92 ± 1.18	<0.001
CCTA interval, years	6.15 (4.10, 8.25)	5.18 (3.33, 7.27)	6.52 (4.67, 8.59)	<0.001
CACS	64.90 (11.30, 158.80)	68.40 (13.10, 163.60)	58.90 (10.00, 157.00)	0.388
Cardiovascular risk factors, *n* (%)				
Hypertension	380 (83.89%)	110 (83.33%)	270 (84.11%)	0.949
Dyslipidemia	144 (31.79%)	50 (37.88%)	94 (29.28%)	0.094
Diabetes Mellitus	185 (40.84%)	54 (41.22%)	131 (40.68%)	>0.999
Current smoker	127 (28.04%)	48 (36.36%)	79 (24.61%)	0.016
Drink	78 (17.22%)	23 (17.42%)	55 (17.13%)	>0.999
Family history of CAD	27 (5.96%)	13 (9.85%)	14 (4.36%)	0.043
Medication history, *n* (%)				
Antiplatelet agents	214 (47.24%)	64 (48.48%)	150 (46.73%)	0.813
Antihypertensive drugs	363 (80.13%)	104 (78.79%)	259 (80.69%)	0.741
Antidiabetic drugs	165 (36.42%)	50 (37.88%)	115 (35.83%)	0.760
Lipid-lowering drugs	217 (47.90%)	61 (46.21%)	156 (48.60%)	0.720
Insulin injection	38 (8.39%)	14 (10.61%)	24 (7.48%)	0.365
Laboratory findings				
TG, mmol/L	1.67 ± 1.15	1.65 ± 1.12	1.68 ± 1.16	0.845
TC, mmol/L	4.56 ± 1.02	4.74 ± 1.06	4.50 ± 1.00	0.027
Glu, mmol/L	6.27 ± 2.05	6.57 ± 2.47	6.14 ± 1.84	0.072
LDL-C, mmol/L	2.90 ± 1.01	3.08 ± 0.91	2.82 ± 1.05	0.011
HDL-C, mmol/L	1.18 ± 0.33	1.17 ± 0.37	1.18 ± 0.311	0.887
hs-CRP, mg/L	1.80 (1.20, 6.97)	2.17 (1.28, 8.76)	1.77 (1.20, 6.46)	0.087
HbA1c, %	6.66 ± 1.34	6.96 ± 1.62	6.53 ± 1.19	0.007
PLT, ×10⁹/L	213.83 ± 59.41	217.40 ± 53.98	212.36 ± 61.52	0.387
Hb,g/L	138.72 ± 15.95	140.56 ± 15.86	137.96 ± 15.95	0.115
WBC, ×10⁹/L	6.70 ± 1.84	6.70 ± 1.88	6.71 ± 1.82	0.986
Creatinine, µmol/L	78.55 ± 19.51	80.54 ± 24.35	77.73 ± 17.10	0.229
UA, µmol/L	355.94 ± 86.97	360.50 ± 84.60	354.06 ± 87.99	0.468
cTnI, μg/L	0.01 (0.01, 0.03)	0.01 (0.00, 0.04)	0.01 (0.01, 0.03)	0.362
BNP, Pg/Ml	81.01 (40.07, 167.70)	70.79 (32.00, 174.00)	83.49 (43.62, 161.20)	0.988

Values are mean ± SD, *n* (%), or median (Q1–Q3).SBP, systolic blood pressure; DBP, diastolic blood pressure; CACS, coronary artery calcium score; TG, triglyceride; TC, total cholesterol; Glu, glucose; LDL-C, low-density lipoprotein cholesterol; HDL-C, high-density lipoprotein cholesterol; hs-CRP, high-sensitivity c-reactive protein; HbA1c, hemoglobin A1c; PLT, platelet; Hb, hemoglobin; WBC, white blood cell; UA, uric acid; cTnI, cardiac troponin I; BNP, B-type natriuretic peptide.

### 3.2 Baseline CCTA and plaque characteristics

Baseline plaque characteristics are summarized in Table 2. Compared with normal Lp(a) group, the elevated Lp(a) group had significantly higher %DS, longer plaque lengths, and smaller minimum lumen areas (*p* < 0.001 for all). Quantitatively, the elevated Lp(a) group also demonstrated greater absolute TPV, FV, FFV, and LAPV (*p* < 0.05 for all). Patients with elevated Lp(a) exhibited a higher proportion of non-calcified volume (*p* < 0.001).

**Table 2:** The coronary plaque and its components at baseline as revealed by CCTA stratified according to the Lp (a) level.

Variables	Overall (*n* = 453)	Elevated group (*n* = 132)	Normal group (*n* = 321)	*p*
PL, mm	21.91 (11.31, 38.88)	31.46 (15.72, 48.39)	18.38 (10.05, 34.64)	<0.001
%DS, %	49 (24, 59)	54 (25, 70)	35 (21, 55)	<0.001
MLA, mm^2^	4.39 (2.97, 6.27)	3.60 (2.52, 5.33)	4.67 (3.17, 6.67)	<0.001
TPV, mm^3^	84.47 (42.37, 153.94)	104.45 (58.02, 502.50)	75.55 (37.43, 145.39)	<0.001
LAPV, mm^3^	4.19 (0.93, 10.53)	6.00 (2.15, 15.70)	3.75 (0.37, 8.68)	0.002
FFV, mm^3^	23.41 (8.56, 51.17)	31.83 (13.72, 86.04)	20.08 (5.84, 44.28)	<0.001
FV, mm^3^	22.18 (12.06, 41.45)	29.25 (16.16, 56.92)	20.51 (10.82, 36.94)	<0.001
CV, mm^3^	17.56 (3.78, 51.05)	19.07 (4.66, 50.74)	17.31 (3.77, 51.11)	0.795
NCV, mm^3^	51.78 (24.29, 105.98)	71.44 (35.81, 167.52)	46.16 (20.49, 92.10)	<0.001
CVR, %	26.59 (6.88, 53.37)	18.57 (3.77, 42.79)	30.98 (9.03, 59.46)	<0.001
FVR, %	28.84 (22.89, 34.15)	29.89 (24.48, 34.37)	28.57 (22.55, 33.94)	0.771
FFVR, %	32.26 (12.88, 48.77)	40.09 (23.07, 53.86)	28.92 (10.03, 46.19)	<0.001
LAPVR, %	4.90 (1.36, 9.91)	6.40 (3.13, 10.36)	4.11 (0.65, 9.33)	0.020
NCVR,%	73.37 (37.66, 98.07)	84.78 (48.32, 99.14)	67.55 (34.20, 97.29)	0.006

PL, plaque length; %DS, percentage diameter stenosis; MLA, minimal lumen area; TPV, total plaque volume; LAPV, low-attenuation plaque volume; FFV, fibro-fatty volume; FV, fibrotic volume; CV, calcified volume; NCV, non-calcified volume; CVR, calcified volume ratio; FVR, fibrotic volume ratio; FFVR, fibro-fatty volume ratio; LAPVR, low-attenuation plaque volume ratio; NCVR, non-calcified volume ratio.

Regarding plaque component ratios, the elevated Lp(a) group had significantly higher FFVR and LAPVR, but a lower CVR (*p* < 0.05 for all). No significant difference was found in the FVR between the two groups (*p* = 0.771).

### 3.3 Association between Lp(a), baseline characteristics and plaque progression over time

In unadjusted linear mixed-effects models (Table 3), the effects of Lp(a) group and follow-up time were evaluated for multiple plaque parameters, including PL, TPV, LAPV, FFV, FV, CV, NCV, LAPVR, FFVR, FVR, NCVR, and CVR.

**Table 3:** Unadjusted and adjusted linear mixed models for the association between Lp(a), follow-up time, and plaque characteristics shown for elevated Lp(a) group and normal Lp(a) group.

Type	Unadjusted *β* (95% CI)	*P* value	Adjusted *β* (95% CI)	*p*
PL (mm)				
Lp(a) > 30	11.23 (6.72, 15.75)	<0.001	9.26 (5.72, 12.81)	<0.001
Follow-up time, per year	0.43 (0.15, 0.71)	0.003	−0.89(−1.22, −0.55)	<0.001
Lp(a) > 30 × follow-up time, per year	0.58(−0.19, 1.36)	0.140	0.03(−0.71, 0.77)	0.937
TPV (mm^3^)				
Lp(a) > 30	45.80 (23.39, 68.22)	<0.001	34.35 (17.94, 50.76)	<0.001
Follow-up time, per year	3.61 (2.06, 5.17)	<0.001	−3.90(−5.55, −2.24)	<0.001
Lp(a) > 30 × follow-up time, per year	2.70(−1.58, 6.99)	0.217	−0.39(−4.17, 3.40)	0.841
LAPV (mm^3^)				
Lp(a) > 30	4.37 (2.28, 6.46)	<0.001	3.54 (1.50, 5.58)	<0.001
Follow-up time, per year	−0.33(−0.51, −0.15)	<0.001	−0.30(−0.52, −0.08)	0.008
Lp(a) > 30× follow-up time, per year	0.59 (0.09, 1.09)	0.022	0.55 (0.04, 1.06)	0.036
FFV (mm^3^)				
Lp(a) > 30	24.31 (15.20, 33.42)	<0.001	20.91 (12.09,2 9.72)	<0.001
Follow-up time, per year	−1.70(−2.34, −1.05)	<0.001	−1.72(−2.55, −0.89)	<0.001
Lp(a) > 30× follow-up time, per year	−0.05(−1.83, 1.74)	0.958	−0.34(−2.17, 1.50)	0.720
FV (mm^3^)				
Lp(a) > 30	13.88 (7.55, 20.20)	<0.001	11.15 (5.68, 16.62)	<0.001
Follow-up time, per year	−0.42(−0.89, 0.05)	0.083	−1.81(−2.39, −1.23)	<0.001
Lp(a) > 30× follow-up time, per year	−0.07(−1.37, 1.22)	0.914	−0.64(−1.99, 0.71)	0.356
CV (mm^3^)				
Lp(a) > 30	1.88(−9.70, 13.47)	0.750	−3.15(−7.96, 1.67)	0.201
Follow-up time, per year	5.95 (4.97, 6.93)	<0.001	−0.02(−0.62, 0.58)	0.947
Lp(a) > 30× follow-up time, per year	2.37(−0.31, 5.05)	0.083	0.32(−1.14, 1.77)	0.671
NCV (mm^3^)				
Lp(a) > 30	43.97 (27.55, 60.39)	<0.001	37.25 (21.77, 52.73)	0.001
Follow-up time, per year	−2.44(−3.59, −1.29)	<0.001	−3.80(−5.28, −2.32)	<0.001
Lp(a) > 30× follow-up time, per year	0.38(−2.79, 3.56)	0.813	−0.45(−3.75, 2.85)	0.788
LAPVR (%)				
Lp(a) > 30	1.92 (0.77, 3.07)	0.001	1.58 (0.49, 2.66)	0.005
Follow-up time, per year	−0.43(−0.55, −0.30)	<0.001	−0.22(−0.35, −0.08)	0.002
Lp(a) > 30× follow-up time, per year	0.40 (0.06, 0.74)	0.022	0.45 (0.11, 0.79)	0.010
FFVR (%)				
Lp(a) > 30	8.25 (4.28, 12.22)	<0.001	7.05 (3.49, 10.61)	<0.001
Follow-up time, per year	−2.18(−2.55, −1.81)	<0.001	−1.4(−1.82, −0.97)	<0.001
Lp(a) > 30× follow-up time, per year	−0.27(−1.29, 0.75)	0.605	−0.19(−1.21, 0.83)	0.712
FVR (%)				
Lp(a) > 30	−0.15(−2.17, 1.87)	0.885	−0.37(−2.36, 1.62)	0.714
Follow-up time, per year	−1.48(−1.73, −1.22)	<0.001	−1.22(−1.50, −0.95)	<0.001
Lp(a) > 30× follow-up time, per year	−0.27(−0.96, 0.41)	0.433	−0.18(−0.86, 0.50)	0.612
CVR (%)				
Lp(a) > 30	−10.53(−15.98, −5.07)	<0.001	−8.75(−13.56, −3.94)	<0.001
Follow-up time, per year	4.19 (3.68, 4.71)	<0.001	2.96 (2.38, 3.54)	<0.001
Lp(a) > 30× follow-up time, per year	0.08(−1.32, 1.48)	0.910	−0.10(−1.50, 1.31)	0.893
NCVR (%)				
Lp(a) > 30	10.31 (4.02, 16.60)	0.001	8.73 (3.04, 14.42)	0.003
Follow-up time, per year	−4.21(−4.83, −3.59)	<0.001	−3.00(−3.70, −2.30)	<0.001
Lp(a) > 30× follow-up time, per year	1.27(−0.42, 2.95)	0.141	1.45(−0.25, 3.16)	0.096

Lp(a), lipoprotein(a); PL, plaque length; PV, plaque volume; LAPV, low-attenuation plaque volume; FFV, fibro-fatty volume; FV, fibrotic volume; CV, calcified volume; NCV, non-calcified volume; LAPVR, low-attenuation plaque volume ratio; FFVR, fibro-fatty volume ratio; FVR, fibrotic volume ratio; CVR, calcified volume ratio; NCVR, non-calcified volume ratio.

A significant interaction between Lp(a) group and follow-up time was found for LAPV (*β* = 0.59 mm^3^/year, 95% CI: 0.09–1.09; *p* = 0.022) and LAPVR (*β* = 0.40%/year, 95% CI: 0.06–0.74; *p* = 0.022). This indicates that patients with elevated Lp(a) experienced a greater increase in these measures over time compared with the normal Lp(a) group. Specifically, LAPV increased annually by 0.26 mm^3^ in the elevated Lp(a) group, while decreasing by 0.33 mm^3^ in the normal group. No significant interactions with follow-up time were detected for PL, TPV, FFV, FV, CV, NCV, FFVR, FVR, NCVR, or CVR (all *p* > 0.05).

After adjusting for age, sex, hypertension, hypercholesterolemia, diabetes, smoking status, family history of CAD, TC, HDL-C, LDL-C, CACS, and statin use, the interactions between Lp(a) group and follow-up time for LAPV (*β* = 0.55 mm³/year, 95% CI: 0.04–1.06; *p* = 0.036) and LAPVR (*β* = 0.45%/year, 95% CI: 0.11–0.79; *p* = 0.010) remained significant (Table 3). This result indicates that elevated Lp(a) is independently associated with a greater increase in low-attenuation plaque volume and its proportion over time.

**Figure 2:**
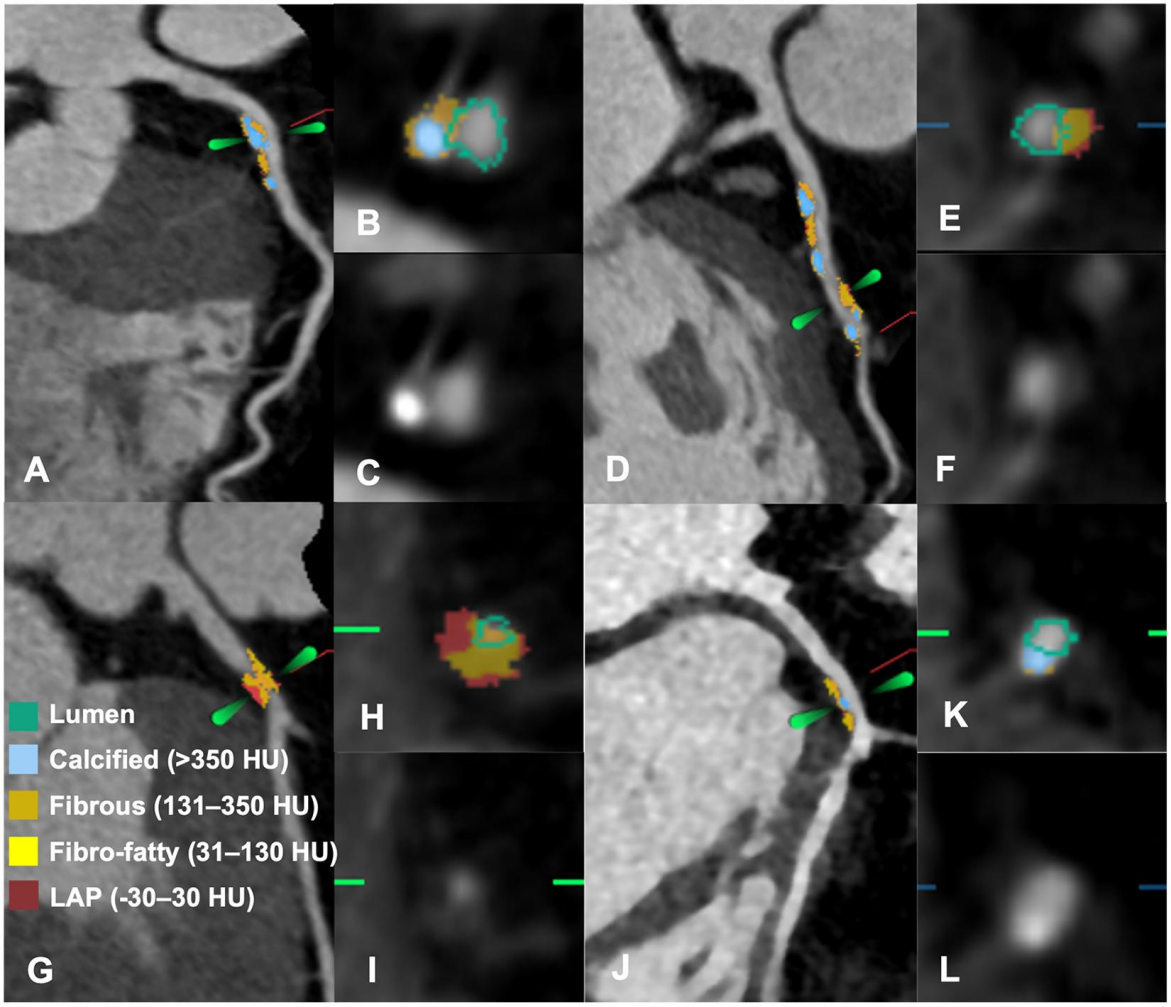
Representative cases of patients with and without plaque progression. **(A–F)** A 59-year-old woman with a baseline Lp (a) concentration of 112 mg/dL. **(A)** Baseline CCTA showed mild stenosis in the proximal LAD. **(B,C)** Cross-sectional reconstruction and component analysis of the target lesion. **(D)** Follow-up CCTA after 5.5 years revealed significant plaque progression, with an increase in LAP volume (7.9 mm^3^) and plaque volume (69.65 mm^3^). **(E,F)** Cross-sectional reconstruction and component analysis of the corresponding plaque. **(G–L)** A 55-year-old man with a baseline Lp(a) concentration of 17.4 mg/dL. **(G)** Baseline CCTA revealed severe stenosis in the proximal LAD. **(H,I)** Cross-sectional reconstruction and plaque component analysis of the target lesion. **(J)** At the 4-year follow-up, CCTA revealed regression of stenosis to a mild degree, with LAP volume and plaque volume decreasing by 20.16 mm^3^ and 85.33 mm^3^, respectively. **(K,L)** Cross-sectional reconstruction and component analysis at follow-up. CCTA, coronary computed tomography angiography; LAD, left anterior descending; LAP, low attenuation plaque; Lp (a), lipoprotein (a); PV, plaque volume.

Elevated Lp(a) was independently associated with greater baseline plaque burden, including longer PL (*β* = 9.26 mm, 95% CI: 5.72–12.81; *p* < 0.001), higher TPV (*β* = 34.35 mm³, 95% CI: 17.94–50.76; *p* < 0.001), NCV (*β* = 37.25 mm³, 95% CI: 21.77–52.73; *p* = 0.001), FFV (*β* = 20.91 mm³, 95% CI: 12.09–29.72; *p* < 0.001), and FV (*β* = 11.15 mm³, 95% CI: 5.68–16.62; *p* < 0.001). An example of Lp(a) and plaque volume change is shown in Figure 2.

Regarding plaque composition ratios, elevated Lp(a) was associated with higher LAPVR (*β* = 1.58%, 95% CI: 0.49–2.66; *p* = 0.005), FFVR (*β* = 7.05%, 95% CI: 3.49–10.61; *p* < 0.001), and NCVR (*β* = 8.73%, 95% CI: 3.04–14.42; *p* = 0.003), and a lower CVR (*β* = −8.75%, 95% CI: −13.56 to −3.94; *p* < 0.001).

A sensitivity analysis using an Lp(a) cutoff of 50 mg/dL yielded consistent associations for baseline plaque burden, with similar trends for LAP progression that did not reach statistical significance (Supplementary Table S1).

### 3.4 Subgroup analysis for the relation of Lp(a) with plaque progression

**Figure 3:**
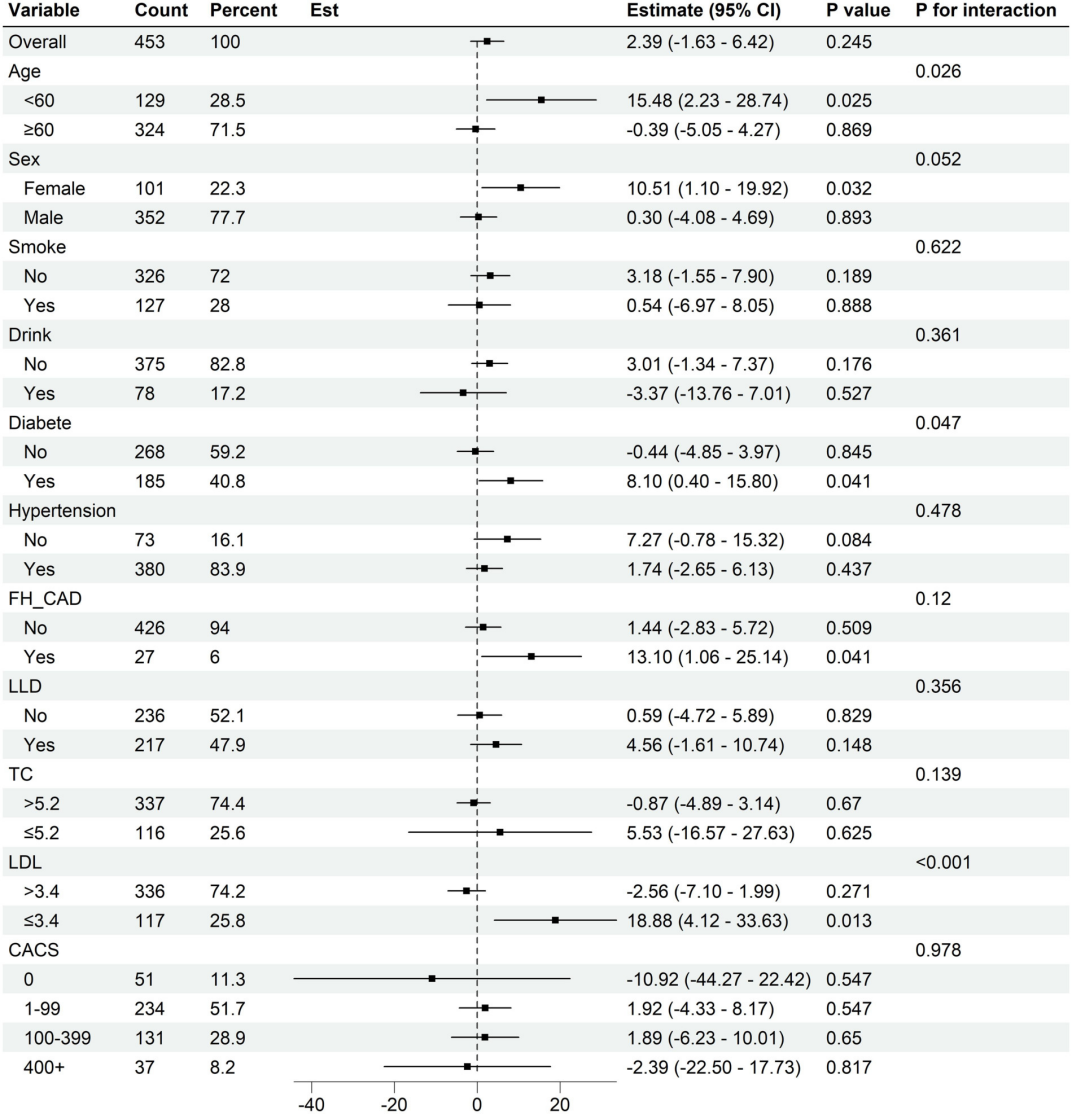
Subgroup analysis for Lp(a) on total plaque volume progression. CI, confidence interval; FH-CAD, family history of coronary artery disease; LLD, lower lipid drug (statin used); LDL-C, low-density lipoprotein cholesterol; TC, total cholesterol; CACS, coronary artery calcium score.

Further evaluation of the association between baseline serum Lp(a) levels and plaque progression across different subgroups (Figure 3, Supplementary Figures S2–S5). A trend toward greater progression in TPV, LAPV, FFV, and FV was observed among patients with diabetes mellitus, family history of CAD, and female sex. A similar pattern of increased plaque progression was also observed in patients aged <60 years and in those with normal lipid profiles (defined as LDL-C ≤ 3.4 mmol/L and TC ≤ 5.2 mmol/L).

Furthermore, patients with diabetes mellitus, female sex, smoking status, drinking, aged <60 years, and normal LDL-C levels (≤3.4 mmol/L) appeared to show greater increasing in CV. Notably, the association between baseline Lp(a) and progression of calcified plaque volume appeared to be more pronounced among patients receiving statin therapy.

## 4 Discussion

In this single-center, retrospective cohort study. We utilized serial quantitative coronary plaque CCTA to provide insights into the association of elevated Lp(a) with plaque progression. Our results demonstrated that elevated baseline serum Lp(a) levels were associated with both a greater baseline plaque burden and a faster rate of progression in LAPV and LAPVR over time. Notably, patients with diabetes mellitus, family history of CAD, and female gender exhibited significantly higher progression in total plaque volume. Particularly in non-calcified plaque volume. Importantly, Lp(a) may represent a significant residual cardiovascular risk factor, even in seemingly low-risk populations such as those aged <60 years and with normal lipid profiles.

CCTA and AI-QCT analysis offers the opportunity to increase accuracy of diagnosis (19, 20), and allow for longitudinal comparable of different plaque types and their progression over time. LAP on CCTA as a quantitative marker of necrotic core was strongest predictor of outcome (10, 11). Yu et al. (21) demonstrated that the concurrence of high Lp(a) and LAP markedly increases myocardial infarction risk. Consistent with many previous studies (22, 23), we observed that elevated Lp(a) levels were associated with accelerated progression of necrotic core, but not with changes in total plaque volume or the evolution of other more stable plaque components. These associations remained significant after adjusting for traditional cardiovascular risk factors, indicating that Lp(a) exerts an independent effect on vulnerable plaque progression. The interaction between Lp(a) group and follow-up time further highlights that high Lp(a) not only correlates with greater initial plaque burden but also modifies the trajectory of plaque evolution, accelerating the growth of necrotic core–rich components. Importantly, our primary findings remained directionally consistent in sensitivity analyses using a more stringent Lp(a) threshold of 50 mg/dL, reinforcing the robust association between elevated Lp(a) and high-risk plaque phenotypes.

This may be partly explained by the fact that Lp(a) is the prominent carrier of pro-inflammatory OxPL, which may partly mediate the atherogenicity of Lp(a) by inducing inflammation and oxidative stress (24). Lp(a) and oxidized phospholipids mediated macrophage apoptosis (25), which is a key component of plaque vulnerability. These phenomena may contribute to accelerated necrotic core formation. As high-risk plaque components primarily develop in the early stages of coronary atherosclerosis (26) and Lp(a) is an inherited risk factor for premature CAD (27). Our data further reaffirm that elevated Lp(a) is associated with necrotic core expansion in atherosclerotic plaques from their initial stages, augmenting the risk of early-onset CAD.

Subgroup analyses suggested that patients with diabetes mellitus, family history of CAD, and female sex tended to exhibit greater progression in total plaque volume, particularly in non-calcified plaque volume. In our study, the population had a mean age of 64.7 years. The incidence of cardiovascular diseases increases in postmenopausal women, potentially attributable to the loss of protective female sex hormones and an increased susceptibility to high-risk plaque features (28–30). Moreover, our study observed that even in seemingly low-risk populations such as those aged <60 years and with normal lipid profiles show a notable trend toward greater progression in total plaque volume especially in non-calcified plaque volume. These patterns are generally consistent with previous finding that Lp(a) may be more strongly associated with non-calcified component or LAP (31), both of which are associated with increased risk of cardiovascular events. These findings may indicate a role of Lp(a) as a significant residual risk factor, partly explaining why some individuals experience plaque progression and cardiovascular events despite achieving normal LDL-C levels (32). However, these subgroup findings should be interpreted with caution and considered hypothesis-generating due to the limited sample size in certain subgroups.

We did not observe significant differences in overall calcification progression between Lp(a) groups, consistent with studies suggesting a weaker association between Lp(a) and coronary artery calcium (CAC) compared to other lipid markers (31, 33–37). Notably, our definition of calcification (>350 HU) likely captured more stable, densely calcified plaque. This may explain the lack of difference, as Lp(a) may be more closely linked to lower-density calcifications (31). Interestingly, the association between Lp(a) and calcification component progression appeared stronger in statin users, aligning with evidence from the PARADIGM study that statins promote plaque transformation toward high-density calcium (38). Taken together, these findings emphasize that composition-specific plaque assessment, rather than CAC scoring alone, may provide a more nuanced understanding of Lp(a)-related risk.

Our findings provide a foundation for future mechanistic studies to explore whether the observed clinical benefits are attributable to LAP regression following Lp(a) lowering. Notably, several clinical trials are currently underway to investigate drugs that lower Lp(a) levels (39, 40)^.^ Our study further substantiates that quantitative CCTA may serve as a valuable tool for monitoring plaque regression in response to these therapies.

This study has several limitations. First, it was a single-center study with a relatively limited sample size which merely consisted of Chinese individuals, and used 30 mg/dL as the cutoff for elevated Lp(a) levels. As different thresholds have been applied in previous studies (21, 22, 41), the results may not be generalized to other populations. Second, the study included only individuals with two or more CCTA examinations, which may introduce selection bias. Asymptomatic patients often lacked follow-up imaging, and those with more severe disease were excluded due to subsequent revascularization after the baseline CCTA—potentially underestimating the true impact of elevated Lp(a) on plaque progression. Third, differences in scanners and acquisition protocols—particularly non-unified tube voltages—may have influenced quantitative analysis. The use of a uniform HU threshold for plaque composition could lead to inaccuracies; low tube voltage increases luminal HU, potentially overestimating calcified plaque and underestimating fibrofatty and necrotic core (42). However, data correlating HU thresholds with the morphology of atherosclerotic plaques are limited. Further research is essential to refine the classification criteria for plaques and to standardize measurement protocols across different scanners. Fourth, unlike some previous studies (23, 41, 43), we did not normalize plaque volumes to vessel volume, which may affect comparability between serial scans. Although we reported both the absolute volumes and volume ratios of each component, consistent results were observed across groups, except for the fibrotic and calcified components. Despite methodological differences, similar results were obtained, further strengthening the evidence that elevated Lp(a) serves as a crucial risk factor. This association is predominantly attributed to the increased proportion of noncalcified components and the progression of LAP, both of which are precursors to subsequent adverse cardiovascular events. Additionally, as quantitative techniques vary in their underlying algorithms, future studies across multiple centers using different scanners and enrolling more diverse populations are needed to explore the generalizability of our findings. Moreover, although CoronaryDoc is a fully automated and deterministic AI-QCT software that generates identical quantitative outputs from the same CCTA dataset regardless of observer or time point—making traditional inter- and intra-observer reproducibility metrics not directly applicable—this study did not perform a separate reproducibility assessment within our own cohort. Future studies incorporating repeated scans or cross-platform validation could further confirm measurement stability across different scanners, populations, and software versions. Finally, we did not assess major adverse cardiovascular events (MACE) such as cardiovascular death or non-fatal myocardial infarction, as coronary plaque progression is a validated surrogate endpoint for future events (44, 45). Nonetheless, the direct relationship between elevated Lp(a) and clinical outcomes should be explored in future multicenter, prospective studies with larger and more diverse populations.

## 5 Conclusion

High serum Lp(a) concentrations are associated with greater baseline coronary plaque burden and accelerated progression of LAP during 6-year follow-up. Beyond traditional clinical risk factors such as family history of CAD, diabetes, smoking, and drinking, our study demonstrates that individuals with elevated Lp(a) levels including those younger than 60 years, female, and those with normal lipid profiles exhibit significant progression in total plaque volume. These findings highlight the importance of early intervention in primary prevention strategies, especially among individuals who might otherwise be classified as low-risk. Future studies investigating the effect of Lp(a)-lowering therapies on coronary plaque burden especially the necrotic core regression is eagerly awaited.

## Data Availability

The original contributions presented in the study are included in the article/Supplementary Material, further inquiries can be directed to the corresponding author.
