# Supplementary material for: Lipoprotein(a) and plaque progression: insights from serial coronary computed tomography angiography and quantitative plaque assessment
Source: Front Cardiovasc Med. 2026 Feb 5;13:1699503. doi: 10.3389/fcvm.2026.1699503 (PMC12916585; doi:10.3389/fcvm.2026.1699503)
Supplement: Supplementary file 1 [file Datasheet1.pdf]

Supplementary Materials

1 CCTA Imaging

Scanner program:Before scanning, Patients were administered sublingual nitroglycerin and , if necessary,oral b-blockade (50 to 100 mg metoprolol) unless contraindicated to achieve a heart rate below 65 beats per minute.CACS was obtained using a noncontrast computed tomography (CT) scan Hence,A bolus of contrast media Iopromide (350 mg I/ml,Ultravist, Jiangsu Hengrui Medicine,Lianyungang, China) with 50–60 mL was injected into the right antecubital vein at the rate of 5 mL/s followed by a 50 mL saline bolus chaser at the same flow rate to provide contrast enhancement. Test Bolus tracking was utilized to optimize the timing of contrast-enhanced imaging,Thus,a region of interest (ROI) was precisely positioned on the descending aorta at the level of the four-chamber heart view,Upon reaching an attenuation threshold of 100 Hounsfield units (Hu), the system was programmed to initiate the contrast-enhanced scan after a delay of 6 seconds. The system automatically reconstructs the optimal images both in diastolic and systolic period within a cardiac cycle after the scan is completed.

Scan parameters： At baseline imaging, all patients underwent combined coronary artery calcium scoring (CACS) and CCTA using first-generation dual-source CT (SOMATOM Definition ,Siemens Healthineers, Germany),CACS was obtained using a noncontrast computed tomography (CT) scan (tube voltage, 120 kV; tube current adopts the CARE DOSE 4D automatic adjustment mode) and reconstructed in the

axial plane with 3mm slice width and 1.5mm increment.The CCTA parameters are as follows: tube voltage of 120 kV , tube current adopts the CARE DOSE 4D automatic adjustment mode ,reconstruction matrix = 512×512,rotation time for 0.33s, the collimation width for 64×0.6mm, the slice thickness for 0.75mm with increment of 0.5 mm.At follow-up, patients also underwent CACS and CCTA using a third-generation dual source CT scanner (SOMATOM Force, Siemens Healthineers, Germany).Patients were administered sublingual nitroglycerin and , if necessary,oral b-blockade (50 to 100 mg metoprolol) unless contraindicated to achieve a heart rate below 65 beats per minute.CCTA was performed with automated tube voltage CARE kV (automatically selected according to the body mass index, the range is 70-120 kV) and the tube current adopts the CARE DOSE 4D automatic adjustment mode,the rotation time for 0.25s, the collimation width for 160×0.6mm, the slice thickness for 0.75mm with increment of 0.5 mm.

2 Supplementary Table

In a sensitivity analysis using an Lp(a) cutoff of 50 mg/dL (Table S1), participants with elevated Lp(a) still demonstrated significantly higher baseline plaque burden, including greater TPV (adjusted β = 33.99 mm³, 95% CI: 10.38–57.62; p = 0.005), NCV (adjusted β = 33.54 mm³, 95% CI: 11.15–55.94; p = 0.003), and FFV (adjusted β = 18.06 mm³, 95% CI: 5.30–30.83; p = 0.006). Although the interaction term for LAPV progression did not reach statistical significance (β = 0.55 mm³/year, 95% CI: –0.12 to 1.23; p = 0.109), the effect direction was consistent with the primary analysis, supporting the robustness of our main findings.

Table S1. Sensitivity analysis of plaque characteristics using Lp(a) ≥ 50 mg/dL as the cutoff: results from unadjusted and adjusted linear mixed-effects models.

Type	Unadjusted β (95% CI)	P value	Adjusted β (95% CI)	*p*
**PL (mm)**				
Lp(a)>50	7.29(0.66, 13.92)	0.031	8.22(3.09, 13.36)	0.002
follow-up time, per year	1.11(0.11, 2.10)	0.029	-0.45(-1.42, 0.51)	0.363
Lp(a)>50 × follow-up time, per year	0.66(-0.37, 1.70)	0.208	0.49(-0.49, 1.47)	0.332
**TPV (mm^3)**				
Lp(a)>50	28.46(-4.31, 61.23)	0.089	33.99(10.38, 57.62)	0.005
Follow-up time, per year	5.43(-0.08, 10.94)	0.054	-3.44(-8.37, 1.48)	0.171
Lp(a)>50 × follow-up time, per year	1.67(-4.04, 7.38)	0.567	0.66(-4.34, 5.67)	0.795

**LAPV (mm^3)**				
Lp(a)>50	2.75（-0.32, 5.81）	0.08	3.17(0.22, 6.12)	0.036
Follow-up time, per year	0.26（-0.38, 0.91）	0.42	0.26(-0.40, 0.93)	0.433
Lp(a)>50× follow-up time, per year	0.57(-0.09, 1.24)	0.09	0.55(-0.12, 1.23)	0.109
**FFV (mm^3)**				
Lp(a)>50	15.29(1.85, 28.73)	0.026	18.06(5.30,30.83)	0.006
Follow-up time, per year	-1.34(-0.95, 3.64)	0.252	-1.63(-4.01, 0.77)	0.184
Lp(a)>50× follow-up time, per year	0.44(-1.93, 2.82)	0.716	0.20(-2.22, 2.62)	0.873
**FV (mm^3)**				
Lp(a)>50	10.23(0.98, 19.47)	0.031	12.03(4.17, 19.88)	0.003
Follow-up time, per year	-0.47(-1.19, 2.14)	0.579	-2.32(-4.07, -0.56)	0.010
Lp(a)>50× follow-up time, per year	-0.02(-1.74, 1.71)	0.986	-0.41(-2.20, 1.38)	0.656
**CV (mm^3)**				
Lp(a)>50	0.002(-16.72, 16.72)	0.999	0.17(-6.72, 7.06)	0.962
Follow-up time, per year	6.76(3.31, 10.22)	<0.001	0.35(-02.24, 1.54)	0.717
Lp(a)>50× follow-up time, per year	0.55(-3.03, 4.13)	0.764	0.33(-1.61, 2.27)	0.740
**NCV (mm^3)**				
Lp(a)>50	28.48(4.26, 52.70)	0.022	33.54(11.15, 55.94)	0.003
Follow-up time, per year	-1.40(-5.47, 2.67)	0.502	-3.36(-7.67, 0.94)	0.126
Lp(a)>50× follow-up time, per year	1.16(-3.06, 5.38)	0.590	0.63(-3.73, 4.98)	0.779
**LAPVR (%)**				
Lp(a)>50	0.37(-1.32, 2.06)	0.670	0.31(-1.27, 1.89)	0.699

Follow-up time, per year	-0.08(-0.36, 0.52)	0.728	0.18(-0.63, 0.26)	0.420
Lp(a)>50× follow-up time, per year	0.34(-0.12, 0.80)	0.149	0.39(-0.07, 0.85)	0.098
**FFVR (%)**				
Lp(a)>50	4.58(-1.24, 10.42)	0.124	4.98(-0.19, 10.15)	0.059
Follow-up time, per year	-2.60(1.29, 3.91)	<0.001	-1.85(-3.17, -0.53)	0.006
Lp(a)>50× follow-up time, per year	-0.38(-1.74, 0.98)	0.590	-0.42(-1.77, 0.94)	0.549
**FVR (%)**				
Lp(a)>50	0.16(-2.78, 3.10)	0.916	0.23(-2.63, 3.09)	0.875
Follow-up time, per year	-1.82(0.93, 2.71)	<0.001	-1.55(-2.44, -0.66)	<0.001
Lp(a)>50× follow-up time, per year	-0.34(-1.76, 0.58)	0.473	-0.34(-1.26, 0.59)	0.475
**CVR (%)**				
Lp(a)>50	-5.59(-13.57, 2.39)	0.171	-5.92(-12.88, 1.04)	0.096
Follow-up time, per year	4.46(2.65, 6.27)	<0.001	3.26(1.44, 5.09)	<0.001
Lp(a)>30× follow-up time, per year	0.22(-1.66, 2.09)	0.820	0.27(-1.60, 214)	0.775
**NCVR (%)**				
Lp(a)>50	4.19(4-5.02, 13.40)	0.373	4.31(-3.96, 12.58)	0.307
Follow-up time, per year	-4.11(-6.29, -1.94)	<0.001	-2.94(-5.15, -0.72)	0.009
Lp(a)>50× follow-up time, per year	-0.01(-2.26, 2.25)	0.996	-0.04(-2.32, 2.23)	0.969

Note: Lp(a), lipoprotein(a); PL = plaque length; PV = plaque volume; LAPV = low-attenuation plaque volume; FFV = fibro-fatty volume; FV = fibrotic volume; CV = calcified volume; NCV = non-calcified volume; LAPVR = low-attenuation plaque

volume ratio; FFVR= fibro-fatty volume ratio; FVR = fibrotic volume ratio; CVR = calcified volume ratio; NCVR = non-calcified volume ratio.

3 Supplementary Figures

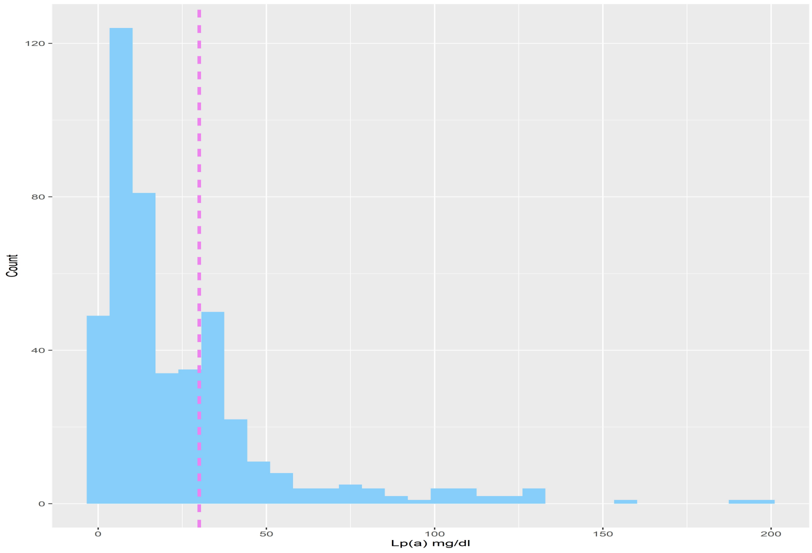

Figure S1. The distribution of LP(a) levels in study cohort

Variable	Count	Percent	Est	Estimate (95% CI)	P value	P for interaction
Overall	453	100		0.64 (0.14 - 1.13)	0.012	
Age						0.006
<60	129	28.5		2.71 (0.81 - 4.61)	0.006	
≥60	324	71.5		0.33 (-0.18 - 0.84)	0.206	
Sex						0.997
Female	101	22.3		0.59 (0.10 - 1.08)	0.021	
Male	352	77.7		0.63 (0.03 - 1.23)	0.041	
Smoke						0.016
No	326	72		0.31 (-0.20 - 0.82)	0.237	
Yes	127	28		1.52 (0.48 - 2.55)	0.005	
Drink						0.833
No	375	82.8		0.70 (0.21 - 1.19)	0.005	
Yes	78	17.2		0.49 (-1.07 - 2.06)	0.539	
Diabete						0.682
No	268	59.2		0.58 (-0.01 - 1.18)	0.056	
Yes	185	40.8		0.80 (-0.06 - 1.66)	0.07	
Hypertension						0.848
No	73	16.1		0.67 (-0.63 - 1.96)	0.318	
Yes	380	83.9		0.59 (0.06 - 1.12)	0.03	
FH_CAD						0.022
No	426	94		0.43 (-0.09 - 0.94)	0.106	
Yes	27	6		2.58 (1.02 - 4.14)	0.003	
LLD						0.398
No	236	52.1		0.78 (0.04 - 1.52)	0.041	
Yes	217	47.9		0.36 (-0.32 - 1.05)	0.3	
TC						0.082
>5.2	337	74.4		0.28 (-0.22 - 0.78)	0.269	
≤5.2	116	25.6		1.09 (-1.35 - 3.53)	0.384	
LDL						0.031
>3.4	336	74.2		0.19 (-0.36 - 0.75)	0.494	
≤3.4	117	25.8		1.65 (0.10 - 3.19)	0.038	
CACS						0.453
0	51	11.3		-0.91 (-6.48 - 4.65)	0.758	
1-99	234	51.7		1.71 (0.69 - 2.72)	0.001	
100-399	131	28.9		1.04 (0.15 - 1.93)	0.023	
400+	37	8.2		-0.12 (-1.69 - 1.44)	0.88	

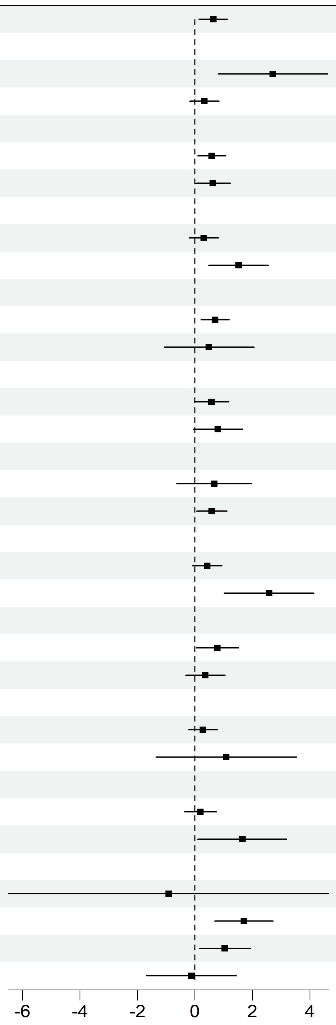

Figure S2. Subgroup analysis for Lp(a) on LAP volume progression. CI,=confidence interval; FH-CAD=family history of coronary artery disease; LLD=lower lipid drug (statin used);,LDL=low-density lipoprotein cholesterol; TC=total cholesterol; CACS=Coronary Artery calcium score；LAP=low-attenuation plaque

Variable	Count	Percent	Est	Estimate (95% CI)	P value	P for interaction
Overall	453	100		-0.35 (-2.07 - 1.37)	0.693	
Age						0.1
<60	129	28.5		-4.09 (-10.63 - 2.45)	0.224	
≥60	324	71.5		-0.49 (-2.37 - 1.40)	0.613	
Sex						0.454
Female	101	22.3		0.78 (-0.64 - 2.20)	0.287	
Male	352	77.7		-0.76 (-2.89 - 1.36)	0.48	
Smoke						0.048
No	326	72		0.88 (-0.85 - 2.61)	0.321	
Yes	127	28		-2.92 (-6.78 - 0.95)	0.142	
Drink						0.048
No	375	82.8		0.26 (-1.42 - 1.93)	0.763	
Yes	78	17.2		-4.87 (-10.66 - 0.92)	0.103	
Diabete						0.265
No	268	59.2		-0.94 (-3.14 - 1.26)	0.403	
Yes	185	40.8		1.19 (-1.53 - 3.92)	0.391	
Hypertension						0.504
No	73	16.1		1.64 (-2.43 - 5.72)	0.434	
Yes	380	83.9		-0.59 (-2.45 - 1.28)	0.536	
FH_CAD						0.029
No	426	94		-1.09 (-2.89 - 0.71)	0.235	
Yes	27	6		6.06 (0.38 - 11.74)	0.045	
LLD						0.383
No	236	52.1		0.20 (-2.12 - 2.52)	0.864	
Yes	217	47.9		-1.33 (-3.92 - 1.26)	0.315	
TC						0.112
>5.2	337	74.4		-1.36 (-3.23 - 0.51)	0.155	
≤5.2	116	25.6		2.58 (-6.39 - 11.55)	0.574	
LDL						0.005
>3.4	336	74.2		-2.57 (-4.65 - -0.48)	0.017	
≤3.4	117	25.8		3.85 (-1.47 - 9.16)	0.159	
CACS						0.439
0	51	11.3		-7.16 (-27.13 - 12.81)	0.511	
1-99	234	51.7		0.55 (-2.86 - 3.95)	0.753	
100-399	131	28.9		0.49 (-3.51 - 4.50)	0.809	
400+	37	8.2		-1.96 (-8.19 - 4.26)	0.538	

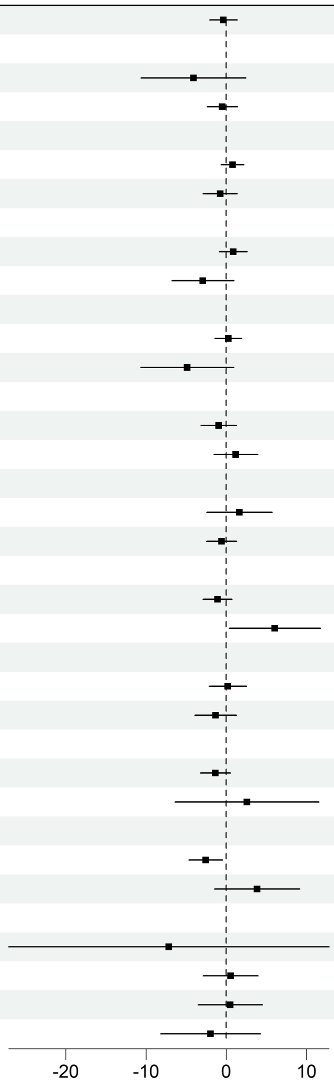

Figure S3. Subgroup analysis for Lp(a) on Fibrofatty component volume progression.

CI=confidence interval; FH-CAD=family history of coronary artery disease;

LLD=lower lipid drug (statin used);,LDL=low-density lipoprotein cholesterol;

TC=total cholesterol; CACS=Coronary Artery calcium score

Variable	Count	Percent	Est	Estimate (95% CI)	P value	P for interaction
Overall	453	100		-0.26 (-1.53 - 1.01)	0.687	
Age						0.835
<60	129	28.5		1.28 (-3.71 - 6.27)	0.616	
≥60	324	71.5		-0.42 (-1.79 - 0.95)	0.546	
Sex						0.269
Female	101	22.3		1.15 (-0.98 - 3.29)	0.294	
Male	352	77.7		-0.62 (-2.11 - 0.88)	0.42	
Smoke						0.097
No	326	72		0.49 (-0.85 - 1.84)	0.472	
Yes	127	28		-1.95 (-4.71 - 0.82)	0.171	
Drink						0.016
No	375	82.8		0.33 (-1.02 - 1.68)	0.632	
Yes	78	17.2		-4.33 (-7.79 - -0.86)	0.017	
Diabete						0.008
No	268	59.2		-1.46 (-2.91 - -0.01)	0.05	
Yes	185	40.8		2.12 (-0.22 - 4.46)	0.078	
Hypertension						0.486
No	73	16.1		1.36 (-1.41 - 4.14)	0.341	
Yes	380	83.9		-0.45 (-1.83 - 0.94)	0.529	
FH_CAD						0.08
No	426	94		-0.69 (-2.04 - 0.66)	0.315	
Yes	27	6		3.56 (-0.37 - 7.50)	0.086	
LLD						0.614
No	236	52.1		-0.75 (-2.58 - 1.07)	0.419	
Yes	217	47.9		-0.05 (-1.89 - 1.78)	0.954	
TC						0.018
>5.2	337	74.4		-1.28 (-2.63 - 0.06)	0.063	
≤5.2	116	25.6		2.84 (-4.12 - 9.79)	0.425	
LDL						0.001
>3.4	336	74.2		-1.70 (-3.18 - -0.21)	0.026	
≤3.4	117	25.8		3.81 (-0.58 - 8.20)	0.091	
CACS						0.984
0	51	11.3		-2.97 (-14.57 - 8.64)	0.619	
1-99	234	51.7		-1.19 (-3.47 - 1.09)	0.307	
100-399	131	28.9		-0.16 (-2.96 - 2.64)	0.91	
400+	37	8.2		-0.26 (-5.80 - 5.29)	0.928	

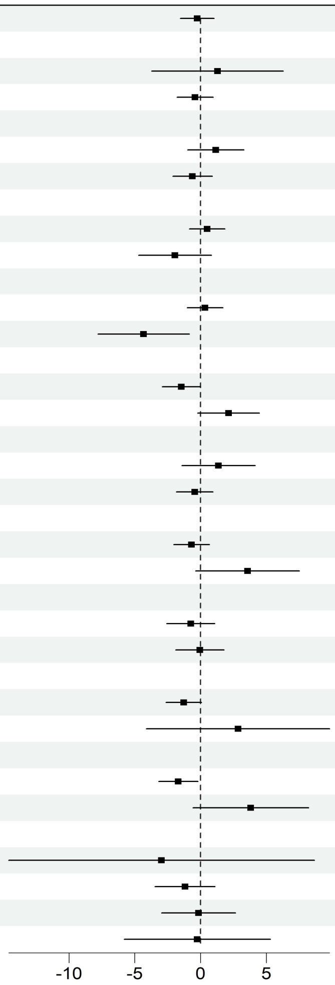

Figure S4. Subgroup analysis for Lp(a) on Fibro component volume progression.

CI=confidence interval; FH-CAD=family history of coronary artery disease;

LLD=lower lipid drug (statin used);,LDL=low-density lipoprotein cholesterol;

TC=total cholesterol; CACS=Coronary Artery calcium score

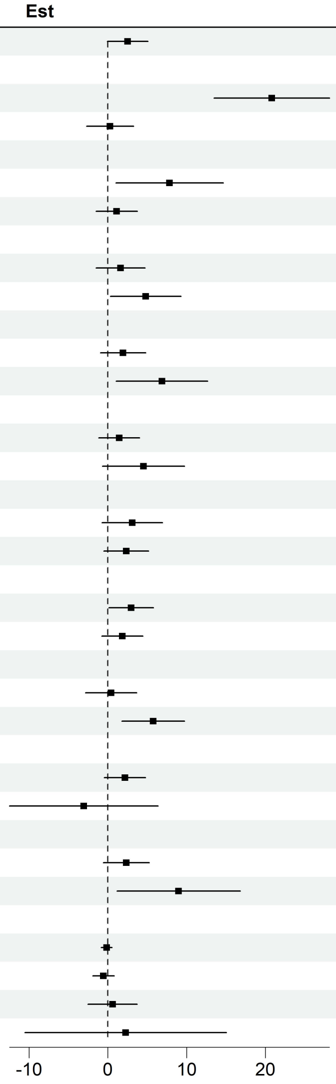

Variable	Count	Percent	Estimate (95% CI)	P value	P for interaction
Overall	453	100	2.51 (-0.07 - 5.08)	0.057	
Age					<0.001
<60	129	28.5	20.80 (13.47 - 28.14)	<0.001	
≥60	324	71.5	0.28 (-2.71 - 3.26)	0.856	
Sex					0.044
Female	101	22.3	7.83 (1.01 - 14.65)	0.027	
Male	352	77.7	1.11 (-1.51 - 3.73)	0.407	
Smoke					0.277
No	326	72	1.62 (-1.50 - 4.73)	0.31	
Yes	127	28	4.81 (0.33 - 9.29)	0.037	
Drink					0.251
No	375	82.8	1.93 (-0.93 - 4.79)	0.187	
Yes	78	17.2	6.86 (1.05 - 12.66)	0.023	
Diabete					0.277
No	268	59.2	1.44 (-1.16 - 4.03)	0.278	
Yes	185	40.8	4.52 (-0.68 - 9.73)	0.09	
Hypertension					0.813
No	73	16.1	3.09 (-0.75 - 6.92)	0.122	
Yes	380	83.9	2.33 (-0.51 - 5.17)	0.109	
FH_CAD					0.806
No	426	94	2.96 (0.14 - 5.77)	0.04	
Yes	27	6	1.83 (-0.77 - 4.43)	0.181	
LLD					0.045
No	236	52.1	0.41 (-2.84 - 3.66)	0.806	
Yes	217	47.9	5.76 (1.79 - 9.73)	0.005	
TC					0.461
>5.2	337	74.4	2.16 (-0.45 - 4.77)	0.106	
≤5.2	116	25.6	-3.07 (-12.49 - 6.36)	0.526	
LDL					0.117
>3.4	336	74.2	2.34 (-0.56 - 5.24)	0.114	
≤3.4	117	25.8	8.98 (1.15 - 16.81)	0.026	
CACS					0.783
0	51	11.3	-0.16 (-0.85 - 0.53)	0.663	
1-99	234	51.7	-0.56 (-1.92 - 0.80)	0.419	
100-399	131	28.9	0.59 (-2.53 - 3.70)	0.712	
400+	37	8.2	2.25 (-10.55 - 15.05)	0.732	

Figure S5. Subgroup analysis for Lp(a) on calcification component volume progression. CI=confidence interval; FH-CAD=family history of coronary artery disease; LLD=lower lipid drug (statin used); LDL=low-density lipoprotein cholesterol; TC=total cholesterol; CACS=Coronary Artery calcium score
